# Gold Foil

**Published:** 1866-12

**Authors:** I. J. Wetherbee

**Affiliations:** Boston


### GOLD FOIL.

BY I. J. WETHERBEE, D. D. S.

In the practice of operative dentistry, gold foil is used to the almost entire exclusion of other materials. Amalgams, fusible materials, or os-artificiel, for the filling of cavities in the teeth, by whomsoever prepared, are admissible in such cases only when the tooth is not worth the *fee* for a gold plug.

In the use of gold foil our patience is often severely taxed by the want of uniformity in its working, so much so, that we feel compelled to try the foil of numerous manufactures, but still with unsatisfactory results. Some styles are hard and brittle at one time, and soft and flexible at another, so that we are kept in a state of *beautiful* uncertainty, at the expense of equanimity of temper and satisfactory operations.

What we require in gold foil is absolute purity from copper and platinum, or any other foreign material, of whatever name or character. When made from gold absolutely pure it will have a uniformity truly refreshing. In folding, it will will not rattle like a brace of swiss bells, nor will it harden when folded and rolled into the form of a pellet, nor will it harden under the pressure of the instrument; also, it will be comparatively soft when annealed or made adhesive by heating. It will work as easily as crystal gold.

I have recently obtained a superior foil made by Dr. S. J. McDougal, of this city, or under his direction, which for purity and softness is superior to any other previously used. Such is its uniformity thus far, that I feel that the debt which I owe the profession requires me to refer to it in this paper. Whoever shall succeed in furnishing the profession with a foil that will lessen our vexations and annoyances, will receive the thanks of every appreciative practitioner of Dentistry.

By a careful comparison of results, I believe we shall arrive at the conclusion, that foil should be non-adhesive as it comes from the manufacturer, and made adhesive by heating when occasion requires. Again, foil for adhesive use should be No. 3, instead of No. 4, as it yields more readily to the pressure of the instrument, admitting a better impaction. The difficulty of filling deep, approximal cavities, either in the incisors or other teeth, will be materially lessened.

Boston, Nov. 15, 1866.

